# “Beyond just the four walls of the clinic”: The roles of health systems caring for refugee, immigrant and migrant communities in the United States

**DOI:** 10.3389/fpubh.2023.1078980

**Published:** 2023-03-30

**Authors:** Seja Abudiab, Diego de Acosta, Sheeba Shafaq, Katherine Yun, Christine Thomas, Windy Fredkove, Yesenia Garcia, Sarah J. Hoffman, Sayyeda Karim, Erin Mann, Kimberly Yu, M. Kumi Smith, Tumaini Coker, Elizabeth Dawson-Hahn

**Affiliations:** ^1^Department of Pediatrics, University of Washington, Seattle, WA, United States; ^2^Independent Consultant, Seattle, WA, United States; ^3^Community Leadership Board, National Resource Center for Refugees, Immigrants and Migrants, San Francisco, CA, United States; ^4^Department of Pediatrics, University of Pennsylvania Perelman School of Medicine, Philadelphia, PA, United States; ^5^Department of Medicine, University of Minnesota, Minneapolis, MN, United States; ^6^Center for Global Health and Social Responsibility, University of Minnesota, Minneapolis, MN, United States; ^7^Seattle Children's Research Institute, Seattle, WA, United States; ^8^School of Nursing, University of Minnesota, Minneapolis, MN, United States; ^9^Division of Epidemiology and Community Health, School of Public Health, University of Minnesota, Minneapolis, MN, United States

**Keywords:** refugee, immigrant, migrant, COVID-19, public health, health system

## Abstract

This article is part of the Research Topic ‘Health Systems Recovery in the Context of COVID-19 and Protracted Conflict’

**Introduction:**

Refugee, immigrant and migrant (hereafter referred to as “immigrant”) communities have been inequitably affected by the COVID-19 pandemic. There is little data to help us understand the perspectives of health systems on their role, in collaboration with public health and community-based organizations, in addressing inequities for immigrant populations. This study will address that knowledge gap.

**Methods:**

This qualitative study used semi-structured video interviews of 20 leaders and providers from health systems who cared for immigrant communities during the pandemic. Interviewees were from across the US with interviews conducted between November 2020–March 2021. Data was analyzed using thematic analysis methods.

**Results:**

Twenty individuals representing health systems participated with 14 (70%) community health centers, three (15%) county hospitals and three (15%) academic systems represented. The majority [16 health systems (80%)] cared specifically for immigrant communities while 14 (70%) partnered with refugee communities, and two (10%) partnered with migrant farm workers. We identified six themes (with subthemes) that represent roles health systems performed with clinical and public health implications. Two foundational themes were the roles health systems had *building and maintaining trust* and *establishing intentionality* in working with communities. On the patient-facing side, health systems played a role in *developing communication strategies* and *reducing barriers to care and support*. On the organizational side, health systems collaborated with public health and community-based organizations, in *optimizing pre-existing systems* and *adapting roles* to evolving needs throughout the pandemic.

**Conclusion:**

Health systems should focus on building trusting relationships, acting intentionally, and partnering with community-based organizations and public health to handle COVID-19 and future pandemics in effective and impactful ways that center disparately affected communities. These findings have implications to mitigate disparities in current and future infectious disease outbreaks for immigrant communities who remain an essential and growing population in the US.

## 1. Introduction

The COVID-19 pandemic has disproportionately affected the health of refugee, immigrant, and migrant communities in the United States (1) (hereafter, “immigrant” communities^1^). Although national-level statistics are sparse, immigrant communities have lower COVID-19 testing prevalence, higher COVID-19 positivity (2), more severe COVID-19 (3, 4) infection and mortality rates twice as high as non-immigrant communities (5, 6). The reasons for these disparities fall into three main categories: community context, health system access, and community experience with government agencies (including public health).

At the community level, multigenerational and higher density housing is a source of collective strength for immigrant communities. However, being near family and social support can increase risk of COVID-19 exposure (7). Moreover, immigrants are often “essential” workers and therefore were excluded from “stay home, stay safe” early in the pandemic (8–10). At the level of health system access, systemic racism and xenophobia prevent equitable access to quality healthcare (11). Immigrants are more likely to be uninsured than their peers (8, 12), and uninsured people are more likely to be hospitalized with COVID-19 infection, adjusting for age, race, ethnicity, and comorbidities (13). At the level of experience with government agencies, fear of legal repercussions from immigration policy is associated with increased risk of COVID-19 infection and decreased healthcare utilization (14, 15). Immigrants also face barriers to inclusion in public health programs, including case investigation and contact tracing (CICT) (16, 17), prompting calls for improved language access within CICT programs and rapid dispersal of culturally and linguistically appropriate public health messaging (14, 18).

Clinicians are often trusted health information messengers (19). When clinicians and health systems gain the trust of immigrant communities, access to healthcare improves (20). Therefore, health systems are critical stakeholders in the public health response to COVID-19 to ensure that programs are effective and inclusive of immigrant communities (21). There is little data, however, describing how health systems serving immigrant communities have navigated the public health response to the COVID-19 pandemic (22). We aim to address this gap in the literature.

## 2. Methods

### 2.1. Study design

We used a qualitative interview study design with data collected for a qualitative needs assessment at the National Resource Center for Refugees, Immigrants and Migrants (Supplementary material). The project was deemed non-human subjects research by the University of Minnesota and exempt by the University of Washington. This exemption status was granted given participants were members of health systems and considered non-vulnerable participants.

### 2.2. Study population

To capture the scope and variation of health system involvement in the public health response, we recruited participants through stratified purposive sampling (23) across specialities, resources, and geography [including all United States Department of Health and Human Services (HHS) regions]. We recruited participants through emails and webforms in existing networks of health care providers, including the Society of Refugee Healthcare Providers, Migrant Clinicians Network, American Academy of Pediatrics Council on Immigrant Child and Family Health, International Rescue Committee, and the Community Leadership Board of NRC-RIM. We sampled health system settings including: academic centers, small rural hospitals, and community health centers. We focused on health systems with established programs serving immigrant communities and anticipated thematic saturation at 20 interviews. Eligible interviewees were individuals from health systems who directly interacted with immigrant communities during the COVID-19 pandemic (e.g., physicians, nurses, administrative staff).

### 2.3. Data collection

We conducted Zoom interviews which lasted up to 60 minutes between 11/11/20 and 3/25/21, using a semi-structured interview guide. The interviews were audio-recorded and professionally transcribed. Interviewees received no compensation. We collected interviewee demographic information including years in practice, education and healthcare setting *via* REDCap electronic data capture tools to ensure we included the right organizational representatives (24, 25).

### 2.4. Data analysis

We developed a preliminary codebook deductively from the interview guide and added inductive codes based on concepts identified in the data. All interviews were coded in Dedoose (26). We held weekly meetings to discuss codebook definitions, emerging codes, and specific excerpts. Finally, we reviewed coded data to identify themes and created a conceptual map of their interrelations based on the thematic analysis methods of Braun and Clarke (27).

## 3. Results

We completed 20 interviews across all HHS regions (Table 1) representing communities from over 30 countries (Table 2). There were interviewees from 14 community health centers (70%), three county hospitals (15%), and three academic health systems (15%). Fourteen of the health systems worked specifically with refugees (70%), two with migrant farmworkers (10%), and 16 with other immigrants (80%). Ten (50%) interviews preceded the Pfizer vaccine emergency use authorization (28). Each interviewee spoke from their own perspective, while also representing their health system and its collective efforts.

**Table 1:** Characteristics of participating organizations (*N* = 20).

	**Health system providers/leaders (%)**
Total number of interviewees	20
**Location, by** HHS region	
1 or 2 (Boston or New York)	3 (15%)
3 or 4 (Philadelphia or Atlanta)	5 (25%)
5 or 6 (Chicago or Dallas)	3 (15%)
7 or 8 (Kansas City or Denver)	2 (10%)
9 or 10 (San Francisco or Seattle)	7 (35%)
**Organizational level** ^*^	
Local (City/County)	20 (20%)
State	0
Regional	0
**Organizational type**	
Community Health Center (could be county, Federally Qualified Health Center [*FQHC]*, etc: HRSA definition)	14 (70%)
County hospital	3 (15%)
Academic health system	3 (15%)
Immigrant-specific organization^**^	4 (20%)
**Populations served** ^***^	
Refugees	14 (70%)
Migrant workers	2 (10%)
Other immigrants	16 (80%)
**Interviewee profession**	
Clinical (physician, nurse practioner, nurse)	16 (80%)
Administrator (director, chief medical officer, manager)	12 (60%)
**Interviewee identifies as a member of an immigrant community**	
Yes	6 (30%)
No	14 (70%)
Interview completed after first COVID vaccine EUA^****^	10 (50%)

* Organization level was categorized as local (e.g., city or county) even if part of a state-wide, regional, or national group when the operational unit that participated in the interview was focused on a local area. For example, an interview focusing on an FQHC's city-wide programming would be categorized as “local” even if the FQHC was part of a state-wide FQHC network.

** We categorized organizations as “refugee, immigrant, migrant-specific” if the organization as a whole or the operational unit within the organization that participated in the interview (e.g., a state refugee health program within a Department of Public Health) focuses specifically on RIM communities.

*** Many organizations work with more than one population.

**** December 11, 2020.

The underline denotes Health and Human Services (HHS) regions, Health Resources and Services Administration.

**Table 2:** Countries of origin of populations served.

**Countries of origin of populations served**	
Afghanistan	India
Algeria	Iraq
Bangladesh	Mexico
Bhutan	Morocco
Bosnia	Myanmar
Burma	Nepal
Cambodia	Nigeria
China	Philippines
Congo	Rohingya
Dominican Republic	Russia
Ecuador	Somalia
El Salvador	South Sudan
Eritrea	Syria
Ethiopia	Ukraine
Guatemala	Vietnam
Honduras	

Communities not identified by country of origin: Asylees, Migrant farm workers, and Hmong.

**Figure 1:**
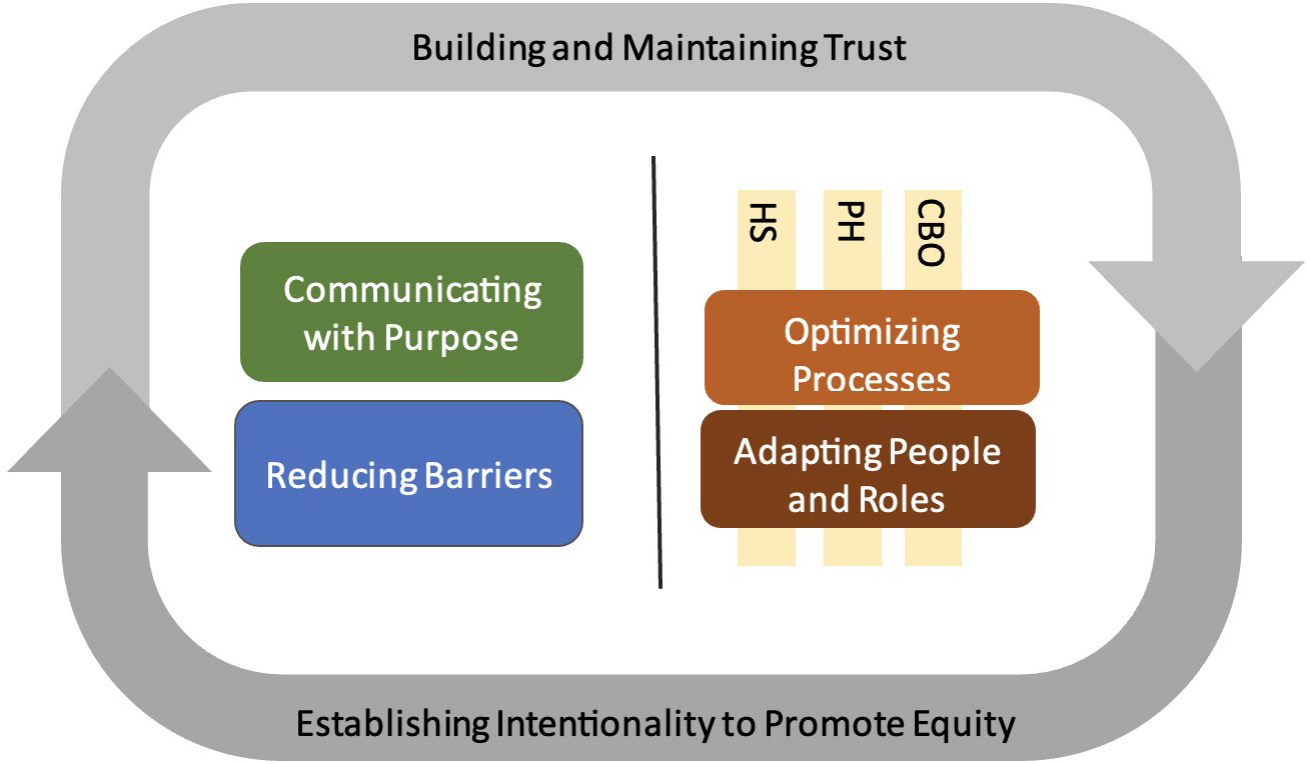
Thematic map demonstrating health system roles on the inward-facing (right of gray line) and outward-facing (left of gray line) aspects of healthcare delivery during the COVID-19 pandemic, enveloped by trust and intentionality. CBO, community-based organization; PH, public health; HS, health systems.

We identified six themes with subthemes: two foundational themes (Table 3) and four operational themes that straddle the inward (organizational/administrative) and outward (patient-facing) roles of health systems (Table 4). The themes are displayed in a conceptual map in Figure 1.

**Table 3:** Foundational themes.

**Establishing intentionality to promote equity**	
Recognizing health disparities and anticipating immigrant-specific needs	“This pandemic has highlighted disparities and magnified them.” HS10
	“There's no question that there's a history of oppression from the period of being a refugee, being resettled and then having to navigate through the social systems in this country and living at the poverty level for a minimum of a decade before you and your children are able to navigate out of it.” HS18
	“For example, yesterday there was a young woman …And she was like, Well, if I test positive, I can't go to work. If I can't go to work, I can't make money and I can't afford housing for my child.” HS02
Acting intentionally to provide equitable care	“Being very intentional… really meeting people where they gather.” HS22
	“In many places, you see people go online and be able to schedule; our communities can't do that… because of the digital divide. So, our staff are basically calling, and we also set up a helpline that they can call, in different languages.” HS22
	“[We] were able to convince all the clinical partners and the county that if a patient showed up at the [health system name] testing center, that they would just get tested without any questions asked … we're one of the only counties in the country that has dramatically oversampled, overtested, our nonwhite population relative to white population.” HS04
	“And we work closely with them, as well, to ensure that there's overinvestment in the limited English proficiency populations for the COVID frontline care team so that they're always placed in the high-risk category, meaning they get the special attention from the outset, and so, in that way, it also works closely with the contact tracing teams.” HS04
**Building and maintaining trust**	
Evaluating immigrant patients' attitudes toward the health system	“[Our] patient population is a population that has a very valid history of not always feeling comfortable with the medical profession.” HS17
	“Patients get tons of terrible bills that don't make any sense, and they're often shafted because they don't speak English… so people are just really hesitant about the healthcare system.” HS16
	“Because a lot of [RIM patients] have a little bit of uncertainty around their immigration status… giving out a lot of information feels pretty uncomfortable.” HS12
	“So many people either don't necessarily give us their correct name and information because of fear of discovery.” HS02
Enhancing patient trust	“The belief that you can all of a sudden show up and say, ‘We're here to help you. Let's give you tests,' doesn't work. And people are still trying to do that, even though it has not worked for a long time.” HS16
	“One is before any pandemic to have partnerships in place so that they can be rapidly operationalized for these sorts of crises, and that means years of building trust and sharing of power is probably the biggest thing.” HS04
	“But I think harnessing relationships that people trust—like it seems that most refugees have pretty good relationships with their resettlement organizations and other community organizations. So, I think there is a good opportunity for those, um, those organizations to really—to really support refugees.” HS02
	“I took it [the vaccine], our CEO took it, and did a video, and [said] ‘Hey, if we turn into zombies tomorrow, we'll let you know,' but we did it in front of everyone, and I think that's what kind of generated this trust.” HS21

Two foundational themes and subthemes with illustrative quotes from health system providers/leaders.

**Table 4:** Outward and inward facing themes.

**Optimizing process**	
Developing new processes through information sharing and merging established systems	“We do public testing. The [redacted city name] Housing Authority is somebody that we've partnered with, and we went to all of their different high-density housing locations like the towers we have here and did different, Saturday morning testing.” HS17
	“Oftentimes there was a lag of five to seven days before the county health department had the information, so what I did is, any time we got a positive, I immediately contacted the county health department… That really helped a lot, because it took away six or seven days that were being missed because of the way the departments communicate with each other.” HS16
Repurposing spaces/facilities	“We've actually had good success in doing school-based testing events, because I think schools are kind of like community health centers. They tend to be trusted spaces for families…”. HS14
	“We're using it in a different way. They've actually been doing smaller groups … that's where they all come together, we provide food, they get organized, and it's kind of the distribution point. So, we've used it a lot, just not in the way that we envisioned.” HS05
**Adapting people and roles**	
Repurposing of roles	“The only way we were really involved was by providing interpreters that they could use to help with the contact tracing, to try to see if people would be willing to give more information from someone who spoke their language. I mean, even though they used the language line, maybe they would recognize they knew this person from within the community.” HS07
Capitalizing on relationships	“But one of the things we've found in our plan for us to be really effective, we felt like we needed to be partnering with somebody who's really already there doing the work.” HS05
	“And we think that leveraging existing community-engaged research partnerships is one way to really adapt quickly to pandemics, not just this one, but in the future....” HS04
	“...these sorts of more grassroots that don't necessarily have much infrastructure but they have really deep social networks.” HS04
	“So, it's been pretty easy to be collaborative because he can say what we can do, he can say what public health can do, and then we have just worked together … A lot of the double roles most of us play, it's a lot less bureaucracy to move and partner. It's a little bit more fluid, I would say. I think that was the biggest thing. Just having him in both camps really helped us be more versatile.” HS12
Supports to staff	“But they just were carrying so much of this. And so, I think they're toward halfway through, and I was just checking in, and they were saying this was totally the space they needed: not just to be there providing the support but needing support themselves so that they could keep continuing.” HS22
**Communicating with purpose**	
Focusing on the message and the messenger	“I think that we have our work cut out for us in terms of … getting people to understand what COVID is, why it's dangerous, and why a vaccine is really important.” HS02
	“...just so that there was a single place for people to at least get some information that was consistent.” HS15
	“Find a spokesperson—who's the spokesperson in the community that people know, and trust, and believe, and have them help get the message across.” HS07
	“When we have our refugees that are resettled, for instance, they typically get a community liaison through [resettlement agency name] as well, so we have a contact there. They use the WhatsApp to help communicate with their families, and then they also already have a rapport with the families as their community partner, so they'll reach out to them.” HS17
Listening to the community	“Communication leaders across nine different languages not only help co-create the messages, but also curate concerns that they're hearing within the refugee communities relative to COVID prevention, testing, and socioeconomic fallout, and bring that back to the group on a daily basis… so that messages can be updated and generated according to what we're hearing on the ground in real time, and also to influence policymakers as to the concerns that are out there from refugee and immigrant communities.” HS04
	“Our center was one of the first to develop and utilize the saliva-based test, so we wanted to do that as a less invasive test offering, because in the beginning we heard from the community leaders that there was a lot of misinformation around the swab, both the invasiveness of the swab, as well as the concern that the swab was actually infecting people and was carrying virus.” HS18
**Reducing barriers**	
Reducing barriers to patient care	“So, we stayed open through the whole pandemic. We thought that was really important and valuable. And part of the reason is we specifically located our clinic in a location that's walkable for the vast majority of the population because so many people in our community lack transportation.” HS15
	“Where we really pride ourselves is we are the communities that we serve, in many cases, so we have staff who are bilingual, bicultural… so the services that we provide are… not through phone connection; it is understanding and very deeply rooted in the cultures that our patients are from.” HS22
	“So, the end of April, we actually had our first what we call community testing day, where we just advertised, we said it's open to the community, anyone, and the idea was barrier-free: you don't need an order, you don't need to be our patient, you don't need insurance.” HS07
	“That's another initiative we have going on, is having a line that actually is specifically for people to get an interpreter and get their phone call triaged within the system.” HS09
	“We've had multiple mask handouts now … to literally hand out masks and place them on every door in an apartment complex.” HS15
Reducing barriers to emergency assistance	“We have what's called the Refugee Drop-In Center, and they were reaching out to some people to see, like, ‘Do you need help applying for unemployment? Do you need help applying for some of those other benefits t hat are available?” HS07
	“Through CARES Act funding through the county, we were basically able to meet any need; it's just a matter of connecting people to that, even things that are very indirectly related to COVID.” HS04
	“One of the things we found out very shortly after the pandemic when people were raiding the stores, is they live on lentils, and they have a special type of rice they like, and those were…you couldn't find them, because people were buying all of the dry goods and storing up, and they couldn't find them, and the prices were going up 400 percent. Because of the hospital having its connections through our nutrition services, and so forth, we found resources to be able to provide every family.” HS05
Reducing barriers for workers and tenants	“And she was afraid to say all these things because she didn't want to get in trouble with her employer and lose her job forever.” HS22
	“I think that's a major issue that pertains to health equity, because if we're talking about patients who are undocumented and don't have a lot of power in the workplace, they need to be supported in this way; whereas people like you or me could potentially work from home and it's a non-issue.” HS14
	“So very, very quickly, and this is something that I think all of us on the COVID team here are so proud of, we made good relationships with those employers from the very get-go, so we were able to go into their employment settings where our patients were most vulnerable and provide them with masks, with sanitization support, with temperature screening equipment, all of those different kinds of things as part of our community liaisons outreach toward them.” HS17
	“We had some employers that, unfortunately, didn't respond as willingly at the beginning, at the onset of COVID. We were able to use some community influence there with our chamber of commerce, with some of our community liaisons and reach out to those places.” HS17

The outward and inward-facing themes (and subthemes) with illustrative quotes from health system providers/leaders.

The foundational themes (theme 1 and 2) are identified as they represented a common thread observed in the operational themes and represented the manner in which the operational themes (themes 3–6) were conducted. The foundational themes represent “how” processes occurred while the operational themes represent “what” processes occurred. We will introduce the foundational themes first to ground the basic approach used to guide actions taken by health system.

In what follows, themes are given in numbered section headings and subthemes are given in italics.

### 3.1. Theme #1: Establishing intentionality to promote equity

Health systems quickly *recognized health disparities specific to immigrant communities* and responded by *acting intentionally to provide equitable care*.

Early on, interviewees discerned that the pandemic “highlighted disparities and magnified them.” One interviewee acknowledged the “oppression from the period of being a refugee, being resettled and then [navigating] social systems... living at the poverty level for a minimum of a decade.” Interviewees noted the disproportionate impact on immigrant communities, including increased COVID-19 exposure risk and more severe disease. They also recognized the challenges communities faced with higher prevalence of comorbidities and lower access to healthcare. Finally, interviewees acknowledged the potential hardships of following public health protocols. One interviewee mentioned a patient who declined COVID testing explaining, “If I test positive, I can't go to work… I can't make money and I can't afford housing for my child.”

Interviewees worked to address health disparities, especially through health system responses centering immigrant communities. One interviewee described tailoring access and “being very intentional… really meeting people where they gather,” while another mentioned strategies to bridge the digital divide. Interviewees also reported how their health systems addressed disparities in testing prevalence. One interviewee said they “convince[d] all the clinical partners and the county that if a patient showed up at the... testing center, that they would just get tested without any questions asked.” Some interviewees described how meeting with multiple regional stakeholders allowed them to shift resources to areas of need.

Interviewees described how they addressed disparities in follow-up with COVID-positive patients. One health system took the unusual step of modifying monitoring protocols to ensure that “limited English proficiency populations… were always placed in the high-risk category, meaning they get the special attention from the outset [by the care team].”

In sum, when interviewees considered how they had interacted with both communities and health systems, they emphasized the importance of maintaining an awareness of disparities and intentionally addressing these disparities through both inward (organizational/administrative) and outward (patient-facing) actions.

### 3.2. Theme #2: Building and maintaining trust

Interviewees *evaluated immigrant patients' attitude toward health systems* and worked to *enhance patient trust*.

Interviewees assessed immigrant patients' attitudes toward health systems and entities perceived to be associated with health systems before taking steps to building trust. Loss of trust is not just historic, as one interviewee explained: patients today “get tons of terrible bills that don't make any sense, and they're often shafted because they don't speak English.” Interviewees understood that certain aspects of the public health response were challenging “because a lot of [immigrant patient communities] have a little bit of uncertainty around their immigration status… giving out a lot of information feels pretty uncomfortable.” Interviewees mentioned reasons for distrust, including “fear of discovery” and legal repercussions like deportation.

Interviewees worked to cultivate the conditions for patient trust using three main strategies: investment over time, harnessing trusted relationships, and transparency. Interviewees recognized that building trust takes time: “The belief that you can all of a sudden show up and say, ‘We're here to help you. Let's give you tests,' doesn't work.” One interviewee stressed that a key to trust-building is having “partnerships in place [that] can be rapidly operationalized for these sorts of crises, and that means years of building trust and sharing power.” Interviewees noted they could support refugees by working with established, trusted entities. “Harnessing relationships that people trust” was key in building trust between health systems and communities. Interviewees acknowledged the fast-changing landscape of COVID-19 misinformation and addressed the need for transparency to build trust. One interviewee discussed vaccination saying, “I took it, our CEO took it, and did a video, and [said] hey, if we turn into zombies tomorrow, we'll let you know… and I think that's what kind of generated this trust.”

As interviewees took steps to understand and ameliorate sources of distrust in health systems, they were able to center their communities in their health system's operational response.

### 3.3. Theme #3: Communicating with purpose

Interviewees played a role in *delivering the public messages used to communicate to communities* in ways and through mediums that were linguistically, culturally, and situationally appropriate while also *listening to the community*.

Interviewees discussed the importance of crafting a succinct, consistent message when information about COVID-19 was rapidly changing and misinformation was widespread. Interviewees tried to match communication strategies to each community's language(s), literacy levels, cultural values, trusted leaders, preferred media, and technology use (e.g., communicating through a resettlement agency that used WhatsApp). They emphasized the messenger: “find a spokesperson… in the community that people know, and trust, and believe.”

Interviewees adopted a bidirectional approach to communication by informing immigrant communities about COVID-19 while gathering their questions and concerns. One interviewee described switching to less invasive saliva-based testing because “we heard from the community leaders that there was a lot of misinformation around … the invasiveness of the swab, as well as the concern that the swab was actually infecting people.”

### 3.4. Theme #4: Reducing barriers

Interviewees recognized their role in encouraging public health measures for communities by reducing *barriers to patient care, barriers to receiving emergency assistance*, and *barriers at work*.

Interviewees reduced barriers to direct patient care related to language, technology, scheduling, transportation, and documentation. One strategy was simply keeping clinics open and communicating with patients. Another strategy was ensuring that patients could navigate services in a language they understood: “Where we really pride ourselves is, we are the communities that we serve, so we have staff who are bilingual, bicultural… the services that we provide are… very deeply rooted in the cultures that our patients are from.” Interviewees also worked to make health measures practical for patients by providing them with supplies like masks and pulse oximeters.

To improve opportunities for testing, health systems offered options that were physically accessible: drive-up, pop-up, or mobile testing near patients' homes. Interviewees reported allowing walk-up testing and scheduling by phone for patients unable to schedule online. Finally, many health systems offered testing without requiring insurance or extensive personal information.

Interviewees reduced barriers to emergency assistance and socioeconomic support by informing immigrant patients about resources, helping them navigate resources, and in some cases by providing direct assistance. Interviewees told immigrant patients about available unemployment and rental assistance and helped them apply. Many interviewees mentioned supplying families with food, striving to make it culturally appropriate whenever possible.

Interviewees recognized that immigrant workers and tenants faced inequitably harsh financial consequences in the event of illness because they often lacked employment/tenant protections. One interviewee said, “patients who are undocumented and don't have a lot of power in the workplace, they need to be supported in this way; whereas, people like you or me could potentially work from home.” Interviewees spoke with patients about work safety concerns, as well as the challenge of navigating public health measures while protecting employment and financial security. One interviewee recalled a patient diagnosed with COVID who “didn't want to get in trouble with her employer and lose her job forever.” Interviewees built relationships through direct communication with employers and landlords *via* letters, phone calls, and in-person visits. One interviewee shared how they provided employers with masks, sanitation support, and thermometers. When employers were unwilling to communicate, the health systems sought out third parties (e.g., boards of health, chambers of commerce, and offices of elected officials) to prompt employer compliance with public health measures.

### 3.5. Theme #5: Optimizing process

Interviewees reported the pandemic created a need to develop new processes by *collaborating and merging established systems with public health and community-based organizations* and by *repurposing spaces*.

Interviewees provided examples of new processes, including a process to implement testing in high-density housing by sharing information with a municipal Housing Authority and Department of Health, and a process to expedite lag times between positive testing and public health CICT through data sharing. Interviewees also discussed combining established systems to provide new services. One example was supporting a community center's testing day by lending health system interpreters to facilitate communication. Collaborating with community partners was also effective: “our community partner organizations let their communities know about [testing] and helped them register, and they were present at the testing site to talk to people.”

The use of physical spaces and facilities was another area where interviewees reported adjusting to better accommodate the needs of immigrant communities. As the country went into lockdown, many public locations were empty, including schools. Schools and school-based health centers, often ideally located near immigrant communities and known as “trusted places for families,” became equipped for testing and other services.

### 3.6. Theme #6: Adapting people and roles

Interviewees reported mobilizing and expanding human resources by collaborating with public health and community-based organizations to *repurpose roles, capitalize on relationships*, and *support staff*.

As the pandemic created a need for new roles, health systems were able to fill gaps by repurposing skilled staff. One interviewee praised a system's resource navigator whose role expanded to finding immigrant-specific resources during the pandemic. Another health system's interpreters conducted contact tracing with public health. In a smaller jurisdiction, one interviewee emphasized the flexibility their health system had because staff also held roles in the public health department: “a lot of double roles most of us play, it's a lot less bureaucracy to move and partner.”

A key to quickly addressing immigrant communities' needs was capitalizing on pre-existing relationships with community-based organizations and other stakeholders. One interviewee developed a relationship with members of the city government: “probably once a month, [we] talk about what's going on, whether it's jobs or neighborhood conditions or health issues.” These relationships ensured information sharing was reciprocal and included diverse perspectives to facilitate fast, effective, and equitable healthcare delivery.

Interviewees lamented the toll the pandemic took on health systems, particularly for employees from immigrant communities. One interviewee expressed concern for struggling staff who “were carrying so much.” As a result, this health system provided extra mental health support and piloted a curriculum for staff support groups. These groups were critical as the staff was “providing the support [to patients] but needing support themselves.”

## 4. Discussion

As the COVID-19 pandemic surged, health systems caring for immigrant communities found themselves responding on two fronts: controlling a new disease and addressing recurrent disparities. Our analysis found health systems addressing both fronts in their outward patient-facing roles as well as their inward-facing, administrative roles. These findings have implications for the remainder of and recovery from this pandemic, future infectious disease outbreaks (i.e., MPX or Monkeypox) and other disaster preparedness efforts as immigrant communities remain an essential and growing population in the US (see action items in Table 5). As it pertains to the COVID-19 pandemic, health systems must repair damage done to their relationships with disproportionately affected immigrant communities. Operational lessons from this pandemic can inform recovery measures that promote resilience in the relationships fostered between health systems and the communities they serve.

**Table 5:** Bringing it home.

**Theme**	**Action items**
Building and maintaining trust	Invest in relationships over time •Partner in activities and events with communities and community organizations over time •Participate in yearly community health-promoting fairs/activities
	Invest in relationships with those trusted by the community •Develop relationships with local businesses, community leaders, religious leaders, trusted advocacy groups, resettlement organizations, etc.
	Model transparency •Record and share on social media videos of trusted individuals receiving vaccines, testing, masking, etc.
Establishing intentionality to promote equity	Providing community-specific support •Set up helpline specifically for communities who prefer a language other than English
	Design intervention for inequity in health outcomes •Designate immigrant communities as High Risk to facilitate close monitoring, contact tracing, and follow-up •Invest in funding for sufficient testing in immigrant communities
Communicating with purpose	Use available media and promote a single, reliable message across all media •Develop a website, WhatsApp group, newsletter, online informational meetings, etc.
	Communicate through trusted sources •Partner with resettlement groups who have frequent touch points with community to communicate information •Partner with religious leaders (priests, imams, rabbis) to communicate information
	Involve communities in message development •Co-create messages with leaders from different communities to ensure consistent messaging across different languages and provide space for communities to participate in messaging
Reducing barriers	Reducing barriers for patients with their employers •To simplify process for providers, develop a formalized process with letter templates for communicating between the health system and employers re: public health recommendations, isolation requirements after test-positivity, quarantine requirements after exposure, etc. •Develop relationships with major employers: assist in developing appropriate screening protocols, provide materials for public health precautions (thermometers, masks, hand sanitizer), provide on-site testing or vaccination
	Reducing barriers to accessing patient care •Routinely evaluate possible barriers to care in the patient population: transportation, insurance, language, work schedules, etc. •Keep local, walkable clinics open during hours that patients are likely to go, and/or take services to patients' communities •Provide translation and interpretation and offer alternatives that don't require literacy or digital literacy •Promote services that do not require information regarding insurance status, immigration status, etc.
	Reducing barriers to emergency assistance •Provide information to patients about emergency food, rental, and other assistance •Develop processes to help patients navigate applications for unemployment and emergency assistance •Work through hospital nutrition services to find wholesale culturally-appropriate foods for community members
Optimizing process	Sharing information to develop new processes •Maintain open lines of communication between health systems and public health to make CICT more efficient and to allow support for patients to start earlier
	Combining systems to develop new processes •Partner with public health and community-based organizations to capitalize on each other's strengths and expertise •Use a clinic's established language database and interpretation services to provide interpretation at public health mass-testing/vaccination events •Use a community organization's preferred mode of communication to distribute informational packets/resources
	Repurposing spaces/facilities •Routinely assess how spaces and facilities are being used, and adjust facility use to fit current or anticipated needs
Adapting people and roles	Flexible/repurposing of roles •Routinely assess how employees and volunteers are being distributed across tasks, and adjust roles to fit current or anticipated needs
	Capitalizing on relationships •Partner with public health and community-based organizations to capitalize on each other's networks and connections
	Supports to staff •Allot time and resources to provide support for staff (apply for funds for mental health resources for staff, provide time and space for support groups, etc.)

Bringing it home: examples of health system interventions by theme.

We found two key themes that underpinned all other themes: intentionality and trust. Health systems are better positioned to plan and execute successful interventions and recovery measures when they understand the diverse situational context and disparities specific to immigrant communities (29–32). By understanding context, health systems can manage their many roles: creating messaging that is linguistically appropriate, recognizing patients' vulnerabilities in the workplace and actively engaging with employers, and identifying areas in the community that are familiar and accessible for testing. Health systems recognized this contextual heterogeneity and adjusted their approaches to the needs and perspectives of their communities.

Just as health systems cannot plan their interventions without cultivating an awareness of burgeoning disparities for immigrant communities, they cannot successfully implement outreach strategies without trust (33–35). For some immigrant communities, concerns involving legal status and the fear of deportation (in the context of the public charge rule) sapped trust in the health system, resulting in fewer immigrants accessing healthcare benefits (10, 36, 37). Partnering with community advocates whose background and connections bring “home” to mind is a proven strategy for building trust throughout the pandemic, and trust supports resilience as partners develop stable relationships that can weather challenges through time (38–41). Our findings support that it takes time and deliberate effort to build trusting relationships with communities and to develop partnerships with community leaders and organizations, particularly before crises (33, 34). Community engagement and trust were vital to the success of the health system interviewees and are critical in preparing for future public health emergencies. While our study was US-focused, similar findings have been shared in studies with immigrant communities globally (42, 43).

We further appreciate the overarching importance of trust and intentionality when we consider healthcare delivery during a crisis. Responding adequately during the pandemic required collaboration between health systems, public health and community organizations/advocates across all processes and interventions. Collaboration fostered sharing data, resources, relationships, and expertise to address needs in critical moments. Health systems were able to use pre-existing processes and resources in combination and to a degree of efficiency that effectively transformed them into new approaches. This was evident through: sharing the benefits of pre-existing trusting relationships, data sharing on COVID-19 cases for geospatial mapping, and sharing established language resources to improve CICT. The benefits of collaboration across sectors to improve public health are well-documented (44, 45), particularly in past crises (46, 47). The cross-sector alignment theory of change developed by the Robert Wood Johnson Foundation emphasizes alignment of public health, health systems and social services, and recognizes the importance of community engagement, without specifying the timing and extent of this engagement (48). Our findings suggest primary, constructive, and enduring collaborations with community-based organizations improved outreach and fostered trust.

This study has limitations worth noting. First, the recruitment method involved self-selection bias and as such, our analysis highlights positive deviance rather than dysfunction. The networks we recruited from and individuals who agreed to our interview represented health systems that identified as caring for immigrant communities; as such, these health systems are among the minority who likely had insight into, investment in, and resources for supporting the varying needs of their communities. This element of selection bias reduces the generalizability of this study. Second, our purposive sampling method limits representation. However, we recruited individuals from a range of health systems that cared for various immigrant communities to capture a diversity in responses. Third, we present the perspectives of health systems without the perspectives of public health and communities within the same jurisdiction, which limits our ability to draw definitive conclusions on the efficacy of collaboration. Nevertheless, this study's strength is the rich descriptions collected from 20 individuals within health systems who interacted with numerous immigrant communities. Future work should center voices from community members to better assess health system efficacy and represent actualized outcomes.

Immigrant communities have been disproportionately harmed by COVID-19. Our findings show that health systems addressed the magnified disparities affecting immigrants by sustaining and reimagining roles to align with the public health response. By focusing on building trust, ensuring intentionality in processes and interventions, and optimizing avenues for collaboration with public health and community partners, health systems can save lives in future public health emergencies.

## Data availability statement

The raw data supporting the conclusions of this article will be made available by the authors, without undue reservation.

## Author contributions

SA and DA had full access to all of the data in the study, take responsibility for the integrity of the data, the accuracy of the data analysis, and involved in drafting the manuscript. ED-H, KYun, and EM were responsible for the study concept, design, and involved in obtaining funding for this project. ED-H, KYun, SA, SH, SK, EM, KYu, CT, DA, WF, KS, and YG were all involved in the acquisition and analysis and interpretation of data. SA, DA, TC, SS, KYun, and ED-H were involved in the critical revision of the manuscript for the important intellectual content. SK provided administrative and technical and material support. ED-H and KYun were involved in study supervision. All authors contributed to the article and approved the submitted version.

## Footnotes

^1^While we will use the term “immigrant” communities, we understand that these communities are each unique with different and rich histories and lived experiences and are not a monolith.

## References

[1] Greenaway C Hargreaves S Barkati S Coyle CM Gobbi F Veizis A. COVID-19: exposing and addressing health disparities among ethnic minorities and migrants. J Travel Med. (2020) 27:taaa113. 10.1093/jtm/taaa113 32706375 PMC7454797

[2] Kim HN Lan KF Nkyekyer E Neme S Pierre-Louis M Chew L. Assessment of disparities in COVID-19 testing and infection across language groups in Seattle, Washington. JAMA Netw Open. (2020) 3:e2021213. 10.1001/jamanetworkopen.2020.21213 32970156 PMC7516622

[3] Wilder JM. The Disproportionate impact of COVID-19 on racial and ethnic minorities in the United States. Clin Infect Dis. (2020) 72:707–9. 10.1093/cid/ciaa959 32648581 PMC7454466

[4] Alcendor DJ. Racial disparities-associated COVID-19 mortality among minority populations in the US. J Clin Med. (2020) 9:E2442. 10.3390/jcm9082442 32751633 PMC7466083

[5] Horner KM Wrigley-Field E Leider JP. A first look: disparities in COVID-19 mortality among US-born and foreign-born Minnesota residents. Popul Res Policy Rev. (2021) 41:465–78. 10.1007/s11113-021-09668-1 34366520 PMC8326639

[6] Garcia E Eckel SP Chen Z Li K Gilliland FD. COVID-19 mortality in California based on death certificates: disproportionate impacts across racial/ethnic groups and nativity. Ann Epidemiol. (2021) 58:69–75. 10.1016/j.annepidem.2021.03.006 33746033 PMC8005258

[7] Brito MO. COVID-19 in the Americas: who's looking after refugees and migrants? Ann Glob Health. (2020) 86:69. 10.5334/aogh.2915 32676298 PMC7333547

[8] Batalova J Hanna M Levesque C. Frequently Requested Statistics on Immigrants Immigration in the United States. migrationpolicy.org. (2021). Available online at: https://www.migrationpolicy.org/article/frequently-requested-statistics-immigrants-and-immigration-united-states (accessed January 28, 2022).

[9] Machado S Goldenberg S. Sharpening our public health lens: advancing im/migrant health equity during COVID-19 and beyond. Int J Equity Health. (2021) 20:57. 10.1186/s12939-021-01399-1 33557854 PMC7868891

[10] Touw S McCormack G Himmelstein DU Woolhandler S Zallman L. Immigrant essential workers likely avoided Medicaid and SNAP because of a change to the public charge rule. Health Aff. (2021) 40:1090–8. 10.1377/hlthaff.2021.00059 34228520 PMC9037600

[11] Mckee MM Paasche-Orlow M. Health literacy and the disenfranchised: the importance of collaboration between limited English proficiency and health literacy researchers. J Health Commun. (2012) 17:7–12. 10.1080/10810730.2012.712627 23030557 PMC3591517

[12] Health Coverage of Immigrants. KFF. (2021). Available online at: https://www.kff.org/racial-equity-and-health-policy/fact-sheet/health-coverage-of-immigrants/ (accessed January 28, 2022).

[13] Killerby ME. Characteristics associated with hospitalization among patients with COVID-19 — Metropolitan Atlanta, Georgia, March–April 2020. MMWR Morb Mortal Wkly Rep. (2020) 69:790–794. 32584797 10.15585/mmwr.mm6925e1 PMC7316317

[14] Clark E Fredricks K Woc-Colburn L Bottazzi ME Weatherhead J. Disproportionate impact of the COVID-19 pandemic on immigrant communities in the United States. PLoS Negl Trop Dis. (2020) 14:e0008484. 10.1371/journal.pntd.0008484 32658925 PMC7357736

[15] Wong TK Cha J Villarreal-Garcia E. The Impact of Changes to the Public Charge Rule on Undocumented Immigrants Living in the U.S. US Immigration Policy Center (2019), p. 14. Available online at: https://usipc.ucsd.edu/publications/usipc-public-charge-final.pdf (accessed March 25, 2022).

[16] Truman BI Tinker T Vaughan E Kapella BK Brenden M Woznica CV. Pandemic influenza preparedness and response among immigrants and refugees. Am J Public Health. (2009) 99:S278–86. 10.2105/AJPH.2008.154054 19461109 PMC4504387

[17] Vaughan E Tinker T. Effective health risk communication about pandemic influenza for vulnerable populations. Am J Public Health. (2009) 99:S324–32. 10.2105/AJPH.2009.162537 19797744 PMC4504362

[18] Maleki P Al Mudaris M Oo KK Dawson-Hahn E. Training contact tracers for populations with limited English proficiency during the COVID-19 pandemic. Am J Public Health. (2021) 111:20–4. 10.2105/AJPH.2020.306029 33326265 PMC7750615

[19] Chung Y Schamel J Fisher A Frew PM. Influences on immunization decision-making among US parents of young children. Matern Child Health J. (2017) 21:2178–87. 10.1007/s10995-017-2336-6 28755045 PMC6026859

[20] Cao QK Krok-Schoen JL Guo M Dong X. Trust in physicians, health insurance, and health care utilization among Chinese older immigrants. Ethn Health. (2023) 28:78–95 10.1080/13557858.2022.2027881 35040724

[21] Haldane V De Foo C Abdalla SM Jung AS Tan M Wu S. Health systems resilience in managing the COVID-19 pandemic: lessons from 28 countries. Nat Med. (2021) 27:964–80. 10.1038/s41591-021-01381-y 34002090

[22] Kim CS Lynch JB Cohen S Neme S Staiger TO Evans L. One academic health system's early (and ongoing) experience responding to COVID-19: recommendations from the initial epicenter of the pandemic in the United States. Acad Med J Assoc Am Med Coll. (2020) 95:1146–8. 10.1097/ACM.0000000000003410 32282371 PMC7176258

[23] Palinkas LA Horwitz SM Green CA Wisdom JP Duan N Hoagwood K. Purposeful sampling for qualitative data collection and analysis in mixed method implementation research. Adm Policy Ment Health. (2015) 42:533–44. 10.1007/s10488-013-0528-y 24193818 PMC4012002

[24] Harris PA Taylor R Thielke R Payne J Gonzalez N Conde JG. Research electronic data capture (REDCap)–a metadata-driven methodology and workflow process for providing translational research informatics support. J Biomed Inform. (2009) 42:377–81. 10.1016/j.jbi.2008.08.010 18929686 PMC2700030

[25] Harris PA Taylor R Minor BL Elliott V Fernandez M O'Neal L. The REDCap consortium: building an international community of software platform partners. J Biomed Inform. (2019) 95:103208. 10.1016/j.jbi.2019.103208 31078660 PMC7254481

[26] Dedoose Version 9,.0.17, Web Application for Managing, Analyzing, Presenting Qualitative Mixed Method Research Data. Los Angeles, CA: SocioCultural Research Consultants, LLC (2021). Available online at: www.dedoose.com (accessed March 21, 2022).

[27] Braun V Clarke V. Using thematic analysis in psychology. Qual Res Psychol. (2006) 3:77–101. 10.1191/1478088706qp063oa

[28] Dwyer C. FDA Authorizes COVID-19 Vaccine For Emergency Use In U.S. NPR. (2020). Available online at: https://www.npr.org/sections/coronavirus-live-updates/2020/12/11/945366548/fda-authorizes-covid-19-vaccine-for-emergency-use-in-u-s (accessed February 15, 2022).

[29] Chang CD. Social determinants of health and health disparities among immigrants and their children. Curr Probl Pediatr Adolesc Health Care. (2019) 49:23–30. 10.1016/j.cppeds.2018.11.009 30595524

[30] Khullar D Chokshi DA. Challenges for immigrant health in the USA-the road to crisis. Lancet Lond Engl. (2019) 393:2168–74. 10.1016/S0140-6736(19)30035-2 30981536

[31] Budiman A. Key findings about U.S. Immigrants. Pew Research Center. Available online at: https://www.pewresearch.org/fact-tank/2020/08/20/key-findings-about-u-s-immigrants/ (accessed February 2, 2022).

[32] Linton JM Green A Council Council on Community Pediatrics. Providing care for children in immigrant families. Pediatrics. (2019) 144:e20192077. 10.1542/peds.2019-2077 31427460

[33] Udow-Phillips M Lantz PM. Trust in public health is essential amid the COVID-19 pandemic. J Hosp Med. (2020) 15:431–3. 10.12788/jhm.3474 32584250

[34] D'Alonzo KT Greene L. Strategies to establish and maintain trust when working in immigrant communities. Public Health Nurs Boston Mass. (2020) 37:764–8. 10.1111/phn.12764 32638421 PMC7484021

[35] Jin K Neubeck L Koo F Ding D Gullick J. Understanding prevention and management of coronary heart disease among Chinese immigrants and their family carers: a socioecological approach. J Transcult Nurs. (2020) 31:257–66. 10.1177/1043659619859059 31268413

[36] McFarling UL. Fearing Deportation, Many Immigrants at Higher Risk of Covid-19 are Afraid to Seek Testing or Care. STAT. (2020). Available online at: https://www.statnews.com/2020/04/15/fearing-deportation-many-immigrants-at-higher-risk-of-covid-19-are-afraid-to-seek-testing-or-care/ (accessed February 14, 2022).

[37] La Rochelle C, Montoya-Williams, D, Wallis, K,. Thawing the Chill from Public Charge Will Take Time Investment. Children's Hospital of Philadelphia PolicyLab. (2021). Available online at: https://policylab.chop.edu/blog/thawing-chill-public-charge-will-take-time-and-investment (accessed February 11, 2022).

[38] Karim N Boyle B Lohan M Kerr C. Immigrant parents' experiences of accessing child healthcare services in a host country: a qualitative thematic synthesis. J Adv Nurs. (2020) 76:1509–19. 10.1111/jan.14358 32189345

[39] Behbahani S Smith CA Carvalho M Warren CJ Gregory M Silva NA. Vulnerable immigrant populations in the New York Metropolitan Area and COVID-19: lessons learned in the epicenter of the crisis. Acad Med. (2020) 95:1827–30. 10.1097/ACM.0000000000003518 32452838 PMC7268828

[40] Alwan RM Kaki DA Hsia RY. Barriers and facilitators to accessing health services for people without documentation status in an anti-immigrant era: a socioecological model. Health Equity. (2021) 5:448–56. 10.1089/heq.2020.0138 34235370 PMC8252901

[41] Nickell A Stewart SL Burke NJ Guerra C Cohen E Lawlor C. Engaging limited English proficient and ethnically diverse low-income women in health research: a randomized trial of a patient navigator intervention. Patient Educ Couns. (2019) 102:1313–23. 10.1016/j.pec.2019.02.013 30772115 PMC8846431

[42] Deal A Hayward SE Huda M Knights F Crawshaw AF Carter J. Strategies and action points to ensure equitable uptake of COVID-19 vaccinations: a national qualitative interview study to explore the views of undocumented migrants, asylum seekers, and refugees. J Migr Health. (2021) 4:100050. 10.1016/j.jmh.2021.100050 34075367 PMC8154190

[43] Nichol AA Parcharidi Z Al-Delaimy WK Kondilis E. Rapid review of COVID-19 vaccination access and acceptance for global refugee, asylum seeker and undocumented migrant populations. Int J Public Health. (2022) 67:1605508. 10.3389/ijph.2022.1605508 36618432 PMC9812946

[44] Rosen M. Impacting quadruple aim through sustainable clinical-community partnerships: best practices from a community-based organization perspective. Am J Lifestyle Med. (2020) 14:524–31. 10.1177/1559827620910980 32922237 PMC7444007

[45] Johnston LM Finegood DT. Cross-sector partnerships and public health: challenges and opportunities for addressing obesity and noncommunicable diseases through engagement with the private sector. Annu Rev Public Health. (2015) 36:255–71. 10.1146/annurev-publhealth-031914-122802 25581149

[46] Millet O Cortajarena AL Salvatella X Jiménez-Barbero J. Scientific response to the coronavirus crisis in Spain: collaboration and multidisciplinarity. ACS Chem Biol. (2020) 15:1722–3. 10.1021/acschembio.0c00496 32584537

[47] Ruckart PZ Ettinger AS Hanna-Attisha M Jones N Davis SI Breysse PN. The flint water crisis: a coordinated public health emergency response and recovery initiative. J Public Health Manag Pract. (2019) 25(Suppl 1):S84–90. 10.1097/PHH.0000000000000871 30507775 PMC6309965

[48] Landers GM Minyard KJ Lanford D Heishman H. A theory of change for aligning health care, public health, and social services in the time of COVID-19. Am J Public Health. (2020) 110:S178–80. 10.2105/AJPH.2020.305821 32663079 PMC7362706

