# Supplementary material for: “Beyond just the four walls of the clinic”: The roles of health systems caring for refugee, immigrant and migrant communities in the United States
Source: Front Public Health. 2023 Mar 30;11:1078980. doi: 10.3389/fpubh.2023.1078980 (PMC10097984; doi:10.3389/fpubh.2023.1078980)
Supplement: Supplementary file 1 [file Data_Sheet_1.docx]

**Supplementary Materials**

Semi-Structured Qualitative Interview Guide

1. Please describe your position, and organization.
2. Can you (briefly) describe the demographics of RIM communities served by your health system/health center?
3. What role has your health system/health center played in case identification and contact tracing (CICT) with RIM communities?
4. Are there any other formal or informal collaborations between your health system/health center and public health authorities, as pertain to the COVID-19 response in RIM communities? (Use prompts as above)
5. In an *ideal scenario* for identifying, contact tracing, and supporting [RIM] patients with COVID, how would you envision your health system/health center engaging with public health and CICT?
6. Assuming a vaccine is found to be safe and effective [AFTER FDA EUA, change to “Now that COVID-19 vaccines have been found to be safe and effective”], what are the most important actions or activities to ensure adequate vaccination coverage among RIM patients in your health system/health center?
7. What discussions, if any, have you/your health system/your health center had with RIM patients about a potential COVID vaccine?
8. What additional information would you like to share about case investigation and contact tracing, potential future vaccination, or the overall COVID response, as it relates to your/your health system’s experience serving RIM communities?
9. Do you or your health system/health center have any materials or tools (websites, data collection tools, etc) that we could share with other organizations serving RIM communities?
10. What additional suggestions or comments do you have for us?
